# Ecthyma gangrenosum due to *Pseudomonas aeruginosa* sepsis as initial manifestation of X-linked agammaglobulinemia: a case report

**DOI:** 10.1186/s12887-020-02436-8

**Published:** 2020-12-01

**Authors:** Haixia Huang, Ke Bai, Yueqiang Fu, Jin Yan, Jing Li

**Affiliations:** 1 Intensive Care Unit, Key Medical Laboratory of Pediatrics, Key Laboratory of Child Development and Disorders, Chongqing Health Bureau, Ministry of Education, Children’s Hospital of Chongqing Medical University, 136#, Zhong Shan 2nd Rord, Yuzhong District Chongqing, People’s Republic of China; 2 Chongqing Key Laboratory of Translational Research for Cancer Metastasis and Individualized Treatment, Chongqing University Cancer Hospital, Chongqing, People’s Republic of China

**Keywords:** Pseudomonas aeruginosa, Ecthyma gangrenosum, X-linked agammaglobulinemia, male infants

## Abstract

**Background:**

X-linked agammaglobulinemia (XLA, OMIM#300,300), caused by mutations in the Bruton tyrosine kinase (*BTK*) gene, is a rare monogenic inheritable immunodeficiency disorder. Ecthyma gangrenosum is a cutaneous lesion caused by *Pseudomonas aeruginosa* that typically occurs in patients with XLA and other immunodeficiencies.

**Case presentation:**

We report the case of a 20-month-old boy who presented with fever, vomiting, diarrhea, and ecthyma gangrenosum. Blood, stool, and skin lesion culture samples were positive for *P. aeruginosa*. A diagnosis of XLA was established, and the c.262G > T mutation in exon 4 of *BTK* was identified with Sanger sequencing. Symptoms improved following treatment with antibiotics and immunoglobulin infusion.

**Conclusions:**

Primary immunodeficiency (i.e., XLA) should be suspected in male infants with *P. aeruginosa* sepsis, highlighting the importance of genetic and immune testing in these patients.

## Background

X-linked agammaglobulinemia (XLA) is a rare inheritable disease characterized by primary immunodeficiency and caused by monogenic mutations in the Bruton tyrosine kinase (*BTK*) gene [1, 2]. The mutations prevent precursor B cells in the bone marrow from forming mature, circulating B-lymphocytes, which results in clinically undetectable levels of all immunoglobulin isotypes. In human subjects with immunodeficiency, XLA has been associated with opportunistic infection by the gram-negative bacterium *Pseudomonas aeruginosa* [3]. Ecthyma gangrenosum is a cutaneous lesion caused by *P. aeruginosa* that requires prompt diagnosis and treatment [4]. Here, we report the case of a 20-month-old boy with XLA and ecthyma gangrenosum caused by *P. aeruginosa* as the initial presenting feature. Sanger sequencing revealed a new hemizygous variant of the X-linked *BTK* gene (c.262G > T in exon 4).

## Case presentation

A 20-month-old boy presented to our institution on April 11, 2018 with a 4-day history of fever reaching 39.5^o^C, vomiting, and diarrhea. The parents noted asymmetrical skin lesions on the patient’s limbs one day prior to admission. He was previously healthy, and had no known history of drug allergies, recent travel, or family history of immunodeficiency. The patient had received age-appropriate live attenuated vaccines and showed no symptoms of discomfort.

**Fig. 1:**
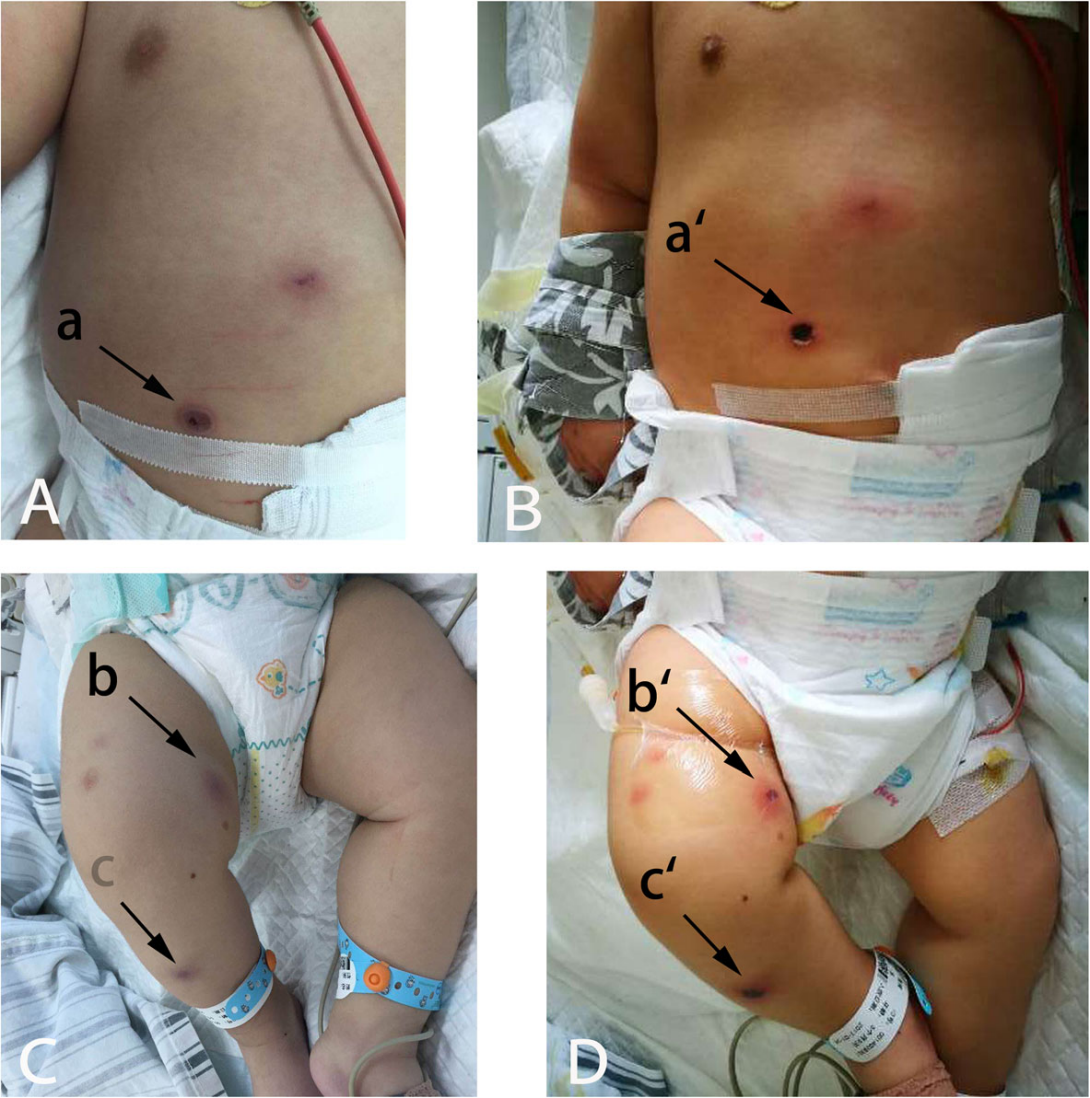
General appearance of the patient showing multiple purple necrotic lesions. (**A**) Day 1, ICU: Initial appearance of the abdominal lesions of ecthyma gangrenosum (a). (**B**) Day 2, ICU: The black central eschar in the lesion was deep seated and large (a`). (**C**) Day 1, ICU: Initial appearance of the lesions over the right lower leg (b&c).(**D**) Day 2, ICU: The erythematous lesions appeared as gangrenous ulcers (b`); The black central eschar was deep seated and large (c`)

On admission, physical examination showed the patient had met typical developmental milestones. He was lethargic and febrile (39.1^o^C); pulse rate was 155/min, respiratory rate was 56/min, and blood pressure was normal. The patient had a bulging anterior fontanelle, pupils were equal and reactive, and there was no abnormality in limb strength. Apparent subcostal retraction was observed. Arterial oxygen saturation (SaO_2_) was 100%. Fine inspiratory rales were present on auscultation. Multiple purple necrotic lesions were visible on the abdomen and the right leg (Fig. 1). Laboratory tests (Table 1) revealed low peripheral white blood cell count 0.26 × 10^9^/L (normal reference values: 4–10 × 10^9^/L) with neutropenia (neutrophils, 0.05 × 10^9^/L; normal reference values: 1.8–6.3 × 10^9^/L ), eosinophils 0.01 × 10^9^/L (normal reference values: 0.02–0.52 × 10^9^/L), lymphocytes 0.16 × 10^9^/L (normal reference values: 1.1–3.2 × 10^9^/L), monocytes 0.04 × 10^9^/L (normal reference values: 0.1–0.6 × 10^9^/L), and elevations in C-reactive protein (CRP, 86 mg/dL; normal reference value: <8.0 ) and procalcitonin (49.04 ng/mL; normal reference value: <2.0).

**Table 1:** Laboratory data

	Day 1	Day3	Day 6	Day 7	Day 8	Day 11	Day 15	Day 40	Reference values
WBC (10^9^/L)	0.26	1.11	4.1	19.44	14.93	26.18	14.67	6.27	4–10
Neutrophils (10^9^/L)	0.05	0.46	2.17	13.61	11.65	22.51	10.71	3.14	1.8–6.3
Eosinophils (10^9^/L)	0.01	0.06	0.21	0.19	0.15	0.00	0.29	0.25	0.02–0.52
Lymphocytes (10^9^/L)	0.16	0.51	0.9	2.53	2.99	2.62	3.08	2.7	1.1–3.2
Monocytes (10^9^/L)	0.04	0.09	0.21	0.78	0.15	0.52	0.59	0.19	0.1–0.6
CRP (mg/dL)	86	60	27	59	83	57	32	< 8	<8.0
Procalcitonin (ng/mL)	49.04	> 100	70.65	42.91	23.09	1.421	0.544	0.044	< 2.0
K^+^ (mmol/L)	3.67	3.17	3.45			3.41	4.36	4.18	3.5–5.5
Na^+^ (mmol/L)	133.3	156.1	138.2			139.3	137.9	141.8	132–149
Cl^−^ (mmol/L)	103.3	97.6	90.6			101.4	104.5	102.3	97–111
Total Ca^2+^ (mmol/L)	2.15	2.64	0.85			2.24	2.46	2.47	2.2-3.0
Mg^2+^ (mmol/L)	0.83	0.77				0.75	0.75	0.93	0.53–1.11
IgG (g/L)		4.94		10.5					2.86–16.8
IgA (g/L)		0.068		< 0.067					0.19–1.75
IgM (g/L)		0.185		0.12					0.43–1.63
Plasma albumin (g/L)		19		37.3					37–52
T lymphocytes (%)		96.48							39–73
B lymphocytes (%)		0.1							7–41

*WBC *white blood cell, *CRP *C-reaction protein

**Fig. 2:**
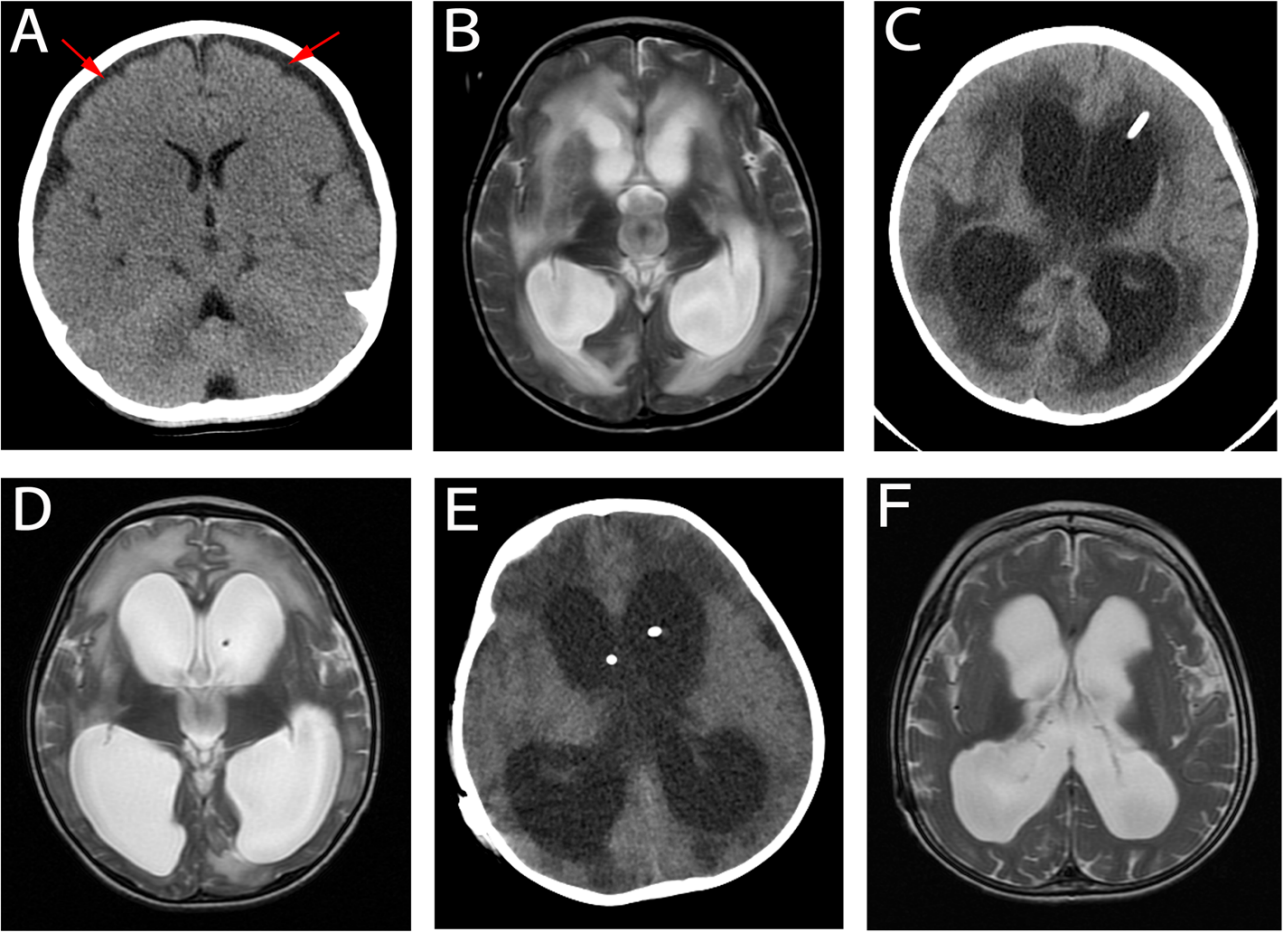
Serial CT and MRI images showing changes in the brain. **a** Day 19 of admission: Lateralcranial CT showing diffuse brain edema, hypodensity of the brain parenchyma, and subdural effusion in the bilateral frontal area (red arrow). **b** Day 25 of admission (one day before Ommaya reservoir implantation): Lateral T2-weighted MRI showing enlarged lateral ventricles and hydrops with peripheral white matter edema. **c** Day 27 of admission (one day after Ommaya reservoir implantation): Lateral cranial CT showing the drainage tube in the anterior horn of the left lateral ventricle. **d** Day 70 of admission (one day before ventriculoperitoneal shunt): Lateral T2-weighted MRI showing hydrocephalus in the ventricles was not obviously aggravated. **e** Day 72 of admission (one day after ventriculoperitoneal shunt): Lateral cranial CT showing the drainage tube in the lateral ventricle. **e** 20 months after discharge: Lateral T2-weighted MRI showing less hydrocephalus and improved interstitial cerebral edema

Ecthyma gangrenosum due to *P. aeruginosa* sepsi*s* was suspected. Intravenous ceftazidime (50 mg/kg, twice a day) was initiated. Four hours after admission, the patient experienced a seizure that was controlled by phenobarbital (5 mg/kg, iv). Two hours later, SaO_2_ decreased to 86%. The patient was transferred to the pediatric intensive care unit (ICU) for mechanical ventilation. One hour after ICU admission, blood pressure was 50/30 mmHg and heart rate was 185/min. The patient received fluid resuscitation and inotropic support. Urine output was < 0.5 mL/kg/h during the 12 hours following ICU admission, and plasma albumin was 19 g/L (normal reference values: 37–52 g/L). Capillary leakage syndrome was considered. Colloids, including albumin, were given intravenously and continuous renal replacement therapy was initiated, but high fever persisted with repeated seizures. Blood, skin lesion, and stool culture samples taken on admission and returned on Day 3 of admission showed *P. aeruginosa* was found in all specimens and was sensitive to meropenem and levofloxacin but resistant to ceftazidime and pieracillin. Ceftazidime was replaced with meropenem (40 mg/kg, q8h) and levofloxacin (5 mg/kg, q12h). On Day 6 of admission, white blood cell count recovered to 4.1 × 10^9^/L and CRP was 27 mg/dL, but enterococcus was found in the cerebrospinal fluid. Meropenem and levofloxacin were replaced with vancomycin (15 mg/kg, q6h) combined with meropenem (40 mg/kg, q8h). On Day 8 of admission, the patient’s body temperature returned to normal. On Day 9 of admission, a cranial computed tomography (CT) scan showed diffuse brain edema (Fig. 2a). Cerebrospinal fluid (CSF) examination revealed protein 5.68 g/L, glucose 2.62 mmol/L (blood glucose, 7.0 mmol /L), total cell numbers 1273*10^6/L, nucleated cells 1238*10^6/L, multinucleated cells 74%, and mononuclear cells 26%. Purulent meningitis was considered, and ceftazidime treatment was continued. Intravenous mannitol (5 ml/kg body weight, q4h) and oxcarbazepine (5 mg/kg body weight, q12h) were given to control intracranial edema and seizures. Over the subsequent three days, the dose of oxcarbazepine was gradually increased to a maximum of 20 mg/kg body weight. The patient was taken off the ventilator. On Day 11 of admission, the patient experienced fever and diarrhea, and *P. aeruginosa* was resistant to meropenem but sensitive to levofloxacin; therefore, meropenem was replaced with levofloxacin. On Day 13 of admission, the patient was transferred back to the general ward; his state of consciousness was evaluated as a light coma. On Day 15 of admission, the patient’s body temperature returned to normal, and his diarrhea was greatly alleviated.

**Fig. 3:**
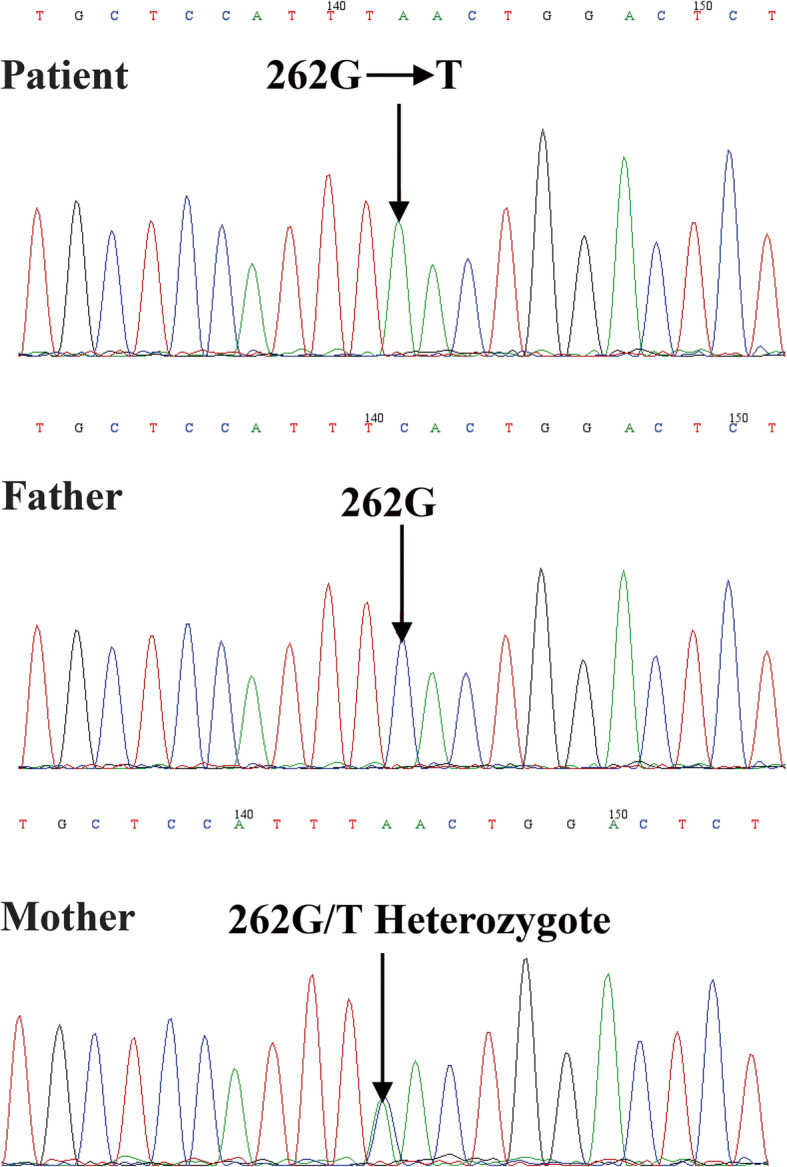
Results of Sanger sequencing for blood samples from the patient (upper panel) and parents (bottom panel)

An immune panel was ordered (Table 1), which showed: plasma IgG 4.94 g/L (normal reference: 2.86–16.8 g/L), IgA 0.068 g/L (normal reference: 0.19–1.75 g/L), and IgM 0.185 g/L (normal reference: 0.43–1.63 g/L). The percentage of T lymphocytes (CD3^+^) was 96.48% (normal reference: 39%-73%) and B-cells (CD3^−^CD19^+^) were absent (0.1%, normal reference: 7%-41%) in peripheral blood. Second generation sequencing of immune-related genes was performed to screen possible mutations; subsequently, Sanger sequencing was applied to verify possible mutations. A hemizygous mutation (c.262G > T) in exon 4 of the X-linked *BTK* gene was found (Fig. 3, upper panel). The patient was diagnosed with XLA, and the mother was identified as a carrier (Fig. 3, bottom panel).

**Fig. 4:**
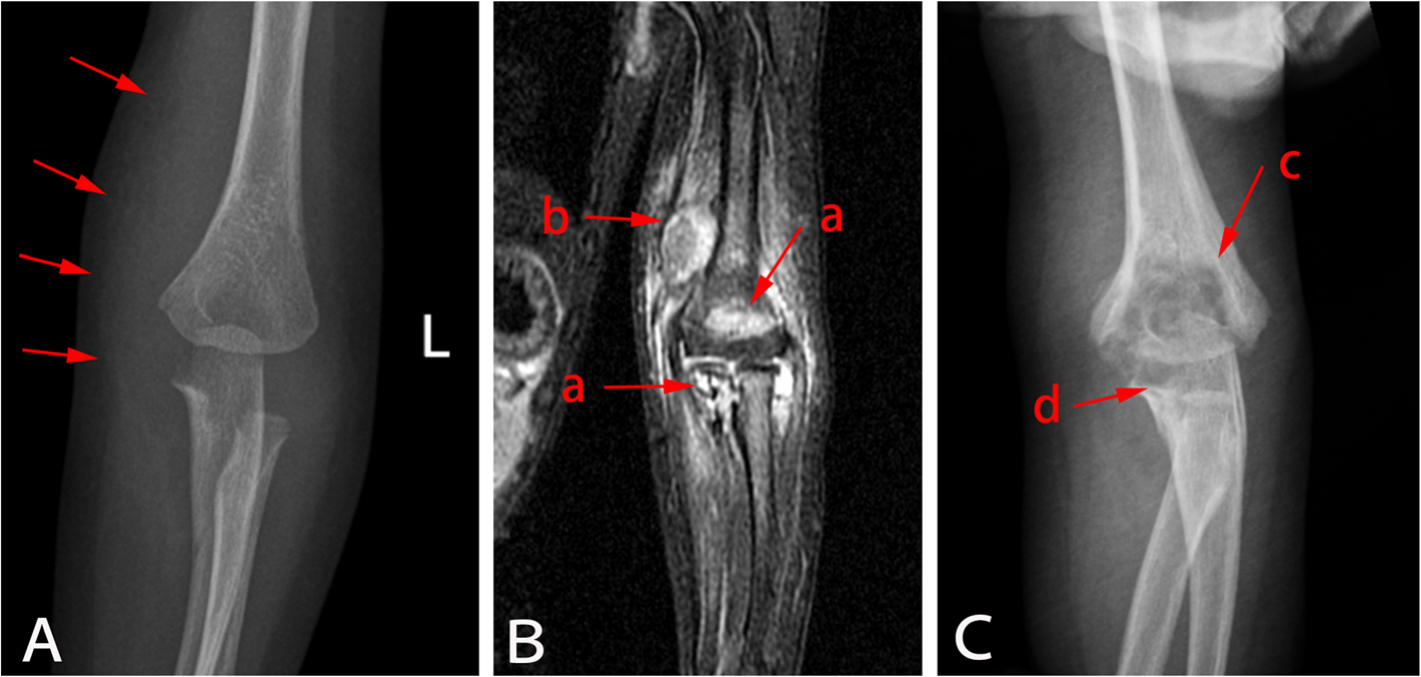
X-ray and MRI images showing changes around the left elbow. (**A**) Day 29 of admission: X-ray showing swelling of soft tissue around the left elbow (arrow). (**B**) Day 47 of admission: Coronal T2-weighted MRI showing patchy hyperintensity in the left humerus and proximal ulna (a) accompanied by soft tissue edema and abscess formation around the elbow joint (b).(**C**) Day 96 of admission: X-ray showing low-density shadows in the left humerus, suggesting bone destruction (c). Similar less severe changes were observed in the proximal left ulna (d). Soft tissue swelling was reduced

On Day 25 of admission, the patient was moderately comatose, spontaneous movements were reduced, and his response to stimulus or pain was poor. Cranial magnetic resonance imaging (MRI) showed severe hydrocephalus, which was managed by Ommaya reservoir implantation (Fig. 2b-c). On Day 70 of admission, repeat cranial MRI showed no significant increase in hydrocephalus, and CSF examination was normal; therefore, a ventriculoperitoneal shunt was inserted (Fig. 2d-e). On Day 29 of admission, the patient’s left elbow became swollen and movement was restricted. X-ray showed soft tissue swelling around the elbow (Fig. 4A). Pyogenic osteomyelitis was considered, and antibiotics (levofloxacin and vancomycin) were continued. On Day 47 of admission, MRI showed bone destruction in the left elbow joint and adjacent soft tissue swelling (Fig. 4B), supporting the diagnosis of pyogenic osteomyelitis. The left elbow remained swollen, but some movement was restored. Repeat culture of blood, stool, and CSF specimens revealed negative results. Considering the condition of the patient, antibiotics were changed to intravenous piperacillin-tazobactam (4:1, 50 mg/kg, q8h) for 3 weeks.

On Day 129 of admission, repeat MRI indicated that hydrocephalus was stable, and the child was discharged from hospital. Monthly intravenous immunoglobulin was prescribed. At the last follow-up on February 10, 2020, the patient remained bed-ridden in a semi-conscious state, with limited responses to stimuli, increased muscle tone, and a normal swallowing reflex.

## Discussion

In the present case, the patient was diagnosed with suspected XLA based on clinical manifestations and results of laboratory tests that showed a life-threatening bacterial infection and severe immunodeficiency, including a marked reduction in all classes of serum immunoglobulins, reduced numbers of B lymphocytes in the peripheral circulation, and severe neutropenia. A diagnosis of XLA was established after the identification of a novel hemizygous variant of the X-linked *BTK* gene on Sanger sequencing.

XLA is a pediatric primary immunodeficiency characterized by a history of recurrent bacterial infections [5]. Encapsulated bacteria, particularly *Streptococcus pneumoniae*, *Haemophilus influenza, Staphylococcus aureus*, and Pseudonmona sp, are the most common bacteria to cause invasive infections in patients with XLA [5, 6]. There is no cure for XLA; therefore, early diagnosis and management to prevent and treat infections is essential.

Patients with XLA typically suffer from recurrent gastrointestinal infections and sino-pulmonary infections such as otitis media, sinusitis, bronchitis, and pneumonia [6]. Patients with XLA rarely present with ecthyma gangrenosum caused by *P. aeruginosa* infection. In the present case, the patient was asymptomatic until 20 months of age, when he presented with *P. aeruginosa* sepsis and ecthyma gangrenosum. *P. aeruginosa* infection has been reported in children with primary immunodeficiency, including Wiskott–Aldrich syndrome, cyclic neutropenia, IRAK-4/MyD-88 deficiency, centromeric region instability, facial anomalies syndrome, and XLA [7–9]. Ecthyma gangrenosum is a key characteristic of *P. aeruginosa* sepsis. Initially it manifests as edematous lesions, then it becomes erythematosus, and finally it evolves into hemorrhagic bullae or gangrenous ulcers with a black central eschar [10].

In 1963, Speirs et al. first described *P. aeruginosa* sepsis and ecthyma gangrenosum as the initial manifestation of XLA [11]. Table 2 summarizes 11 similar cases in the published literature [11–17]. Among these, patients were aged from 6-months to 28-months [11–17]. In only five cases (including the present case), XLA diagnosis was confirmed by sequencing of the *BTK* gene [15–17]. Except for one report that included twins [16], all identified *BTK* mutations were novel, suggesting that sequencing the whole *BTK* gene is more reliable than site specific polymerase chain reaction (PCR) or hybridization to confirm the molecular diagnosis of XLA.

**Table 2:** Summary of cases of *P. aeruginosa* sepsis and ecthyma gangrenosum as the initial manifestation in XLA patients

Year	Cases No.	Exon/intron	Mutation	Age (mos)	Antibiotics	Ref
2020	1	Exon 4	c.262G > T	20	meropenem/levofloxacin	present
2020	2	Exon 10	c.862C > T	7	ceftazidime/gentamicin	[17]
2019	1	-	c.1555T p.H519Y	11	piperacillin-tazobactam /ciprofloxacin	[16]
2018	1	-	c.726dupT; p.Ile243TyrfsTer15	19	meropenem	[15]
2017	1	-	NA	16	meropenem/gentamicin	[14]
1996	2	-	NA	28, 24	ticarcillin/gentamicin ticarcillin/gentamicin	[13]
1991	2	-	NA	6 30	carbenicillin/tobramycin ampicillin/piperacillin/amikacin	[12]
1963	1	-	NA	11	Colomycin	[11]

Intravenous immunoglobulin replacement is standard of care for XLA, but antibiotics were administered in all patients with *P. aeruginosa* infection. Notably, meropenem or gentamicin displayed efficacy in most previous reports (7 cases) [13–15, 17]. The twins with XLA and *P. aeruginosa* infection were initially treated with meropenem [16]; however, antibiotic susceptibility tests of the isolated *P. aeruginosa* strain showed complete resistance; therefore, therapy was changed to piperacillin-tazobactam plus ciprofloxacin. In the present case, although the first antibiotic susceptibility test showed *P. aeruginosa* was sensitive to meropenem, resistance developed, and antibiotic therapy was changed to levofloxacin. These findings confirm that regular monitoring with antibiotic susceptibility tests is essential to determine the effectiveness of antibiotics for *P. aeruginosa* infection in patients with XLA.

## Conclusions

XLA should be considered in previously healthy patients with *P. aeruginosa* sepsis and ecthyma gangrenosum, especially in male infants. Sequencing of the whole *BTK* gene is required to confirm diagnosis, and regular monitoring with antibiotic susceptibility tests is needed to achieve a satisfactory outcome.

## Data Availability

All data generated or analyzed during this study are included in this published article.

## Abbreviations

XLA: X-linked agammaglobulinemia
BTK: Bruton tyrosine kinase
SaO_2_: Oxygen saturation
CRP: C-reactive protein
ICU: Intensive care unit
MRI: Magnetic resonance imaging
CT: Computed tomography
CSF: Cerebrospinal fluid
